# Quality traits and fatty acid composition in meat of Hair Goat and Saanen  ×  Hair Goat (G_1_) crossbred kids fattened in different systems

**DOI:** 10.5194/aab-64-305-2021

**Published:** 2021-07-19

**Authors:** Hacer Tüfekci, Mustafa Olfaz

**Affiliations:** 1 Department of Animal Science, Agricultural Faculty, Yozgat Bozok University, 66100, Yozgat, Turkey; 2 Department of Animal Science, Agricultural Faculty, Ondokuz Mayıs University, 55200, Samsun, Turkey

## Abstract

In this study, meat quality traits and fatty acid compositions of Hair Goat and Saanen × Hair Goat ($G_{1}$) crossbred kids fattened under intensive, semi-intensive and extensive conditions were determined. For meat quality traits, differences in ${\mathrm{pH}}_{24\;h}$, ${\mathrm{pH}}_{45\;\min}$, drip loss, water holding capacity, cooking loss and Warner–Bratzler peak shear force values of the experimental groups were not found to be significant. According to colour measurements at the 0th and 45th minute, the extensive fattening group of Hair Goat kids had greater lightness ($L^{*}$) values and the intensive fattening group of Hair Goat kids had greater redness ($a^{*}$) values. For intensive, semi-intensive and extensive fattening groups of Hair Goat kids, total saturated fatty acid contents of longissimus dorsi (LD) muscle samples were respectively measured as 19.28 %, 23.75 % and 23.35 %. Total monounsaturated fatty acid contents were respectively measured as 67.30 %, 66.22 % and 65.72 %. Total polyunsaturated fatty acid contents were respectively measured as 5.46 %, 3.06 % and 3.16 % and conjugate linoleic acid contents were respectively measured as 0.48 %, 0.55 % and 0.65 %. For intensive, semi-intensive and extensive fattening groups of Saanen × Hair Goat ($G_{1}$) kids, total saturated fatty acid contents of LD muscle samples were respectively measured as 21.01 %; 21.98 %, 19.10 %; total monounsaturated fatty acid contents were respectively measured as 64.04 %, 64.33 %, 52.44 %. Total polyunsaturated fatty acid contents were respectively measured as 3.53 %, 4.89 % and 4.84 % and conjugate linoleic acid contents were respectively measured as 0.52 %, 0.58 % and 0.73 %. It was concluded that the extensive fattening group had greater conjugated linoleic acid contents than the other fattening groups.

## 1 Introduction

Goat meat is an important source of food in developing countries. Goats can efficiently benefit from feed sources in regions with limited feed sources and deficit water resources and offer meat and dairy products most economically (Sandfort, 1982; Upton, 2004; Yalçıntan et al., 2012). In Turkey, goat farming has been traditionally performed in pastures and with local breeds for centuries and provided significant contribution to economy and socio-cultural structure of the goat-farming regions (Bolacalı and Küçük, 2012). There are 11 367 goats in Turkey and with this number, goats have the third place in livestock after sheep and cattle. According to 2019 statistics, 13 % of red meat production of Turkey comes from ovine and 3.5 % of it comes from goat farming (TURKSTAT, 2020).

Considering the incomes earned from goat farming, meat production has an important place compared to other yield parameters. Goat meat with high protein and low fat content plays an important role in human nutrition. Meat quality is designated by genotype, gender, age and feeding conditions (Johnson et al., 1995; Boyazoğlu and Morand-Fehr, 2001). Compared to sheep and cattle meat, goat meat has lower fat and cholesterol content. As well as this, goat meat has greater polyunsaturated fatty acid contents compared to the other ruminants (Mahgoub and Lu, 1998). Previous research conducted with different goat breeds reported differences in the fatty acid composition of the goat breeds and indicated that some breeds were quite rich in unsaturated fatty acids as compared to the others (Brzostowski et al., 2008; Madruga et al., 2009; Banskalieva et al., 2000; Horcada et al., 2012). When the means of production of Turkey were assessed together with the consumption habits of the people, it was seen that current goat meat production levels could be improved. Therefore, further research is needed to improve goat meat production and quality. In the present study, meat quality traits and fatty acid composition of Hair Goat and Saanen × Hair Goat ($G_{1}$) crossbred kids fattened under intensive, semi-intensive and extensive conditions were determined.

## 2 Material and method

Research protocol of the present study was approved by Local Ethical Committee of Ondokuz Mayıs University for Animal Experiments (with the decision number of 2013/67).

Singleton, weaned Hair Goat and Saanen × Hair Goat ($G_{1}$) crossbred male kids at the age of 2.5–3 months were used as the animal material in the present study. The kids were brought to the facility and internal and external parasite checks were performed. Experiments were initiated after 2 weeks of adaptation fattening. In this study experiments were conducted in a 2 × 3 factorial design (Hair Goat and Saanen × Hair Goat ($G_{1}$) crossbred kids × intensive, semi-intensive and extensive) with 10 animals in each group.

**Table 1:** Nutritional composition of feeds used in fattening systems and samples taken from pasture (%).

Nutritional composition	Dry meadow grass	Concentrate feed	Pasture grass
Dry matter	89.40	90.00	92.43
Crude protein	7.09	17.00	12.43
Crude fat	1.25	3.00	2.91
Crude cellulose	34.90	10.00	28.74
Crude ash	8.01	9.00	8.84
Organic matter	81.36	81.00	83.37
ADF	41.58	21.61	31.32
NDF	67.89	42.17	51.27

ADF: acid detergent fibre. NDF: neutral detergent fibre.

Kids in intensive and semi-intensive treatments were penned individually in 1.5 $m^{2}$ pens, while kids in extensive treatment were penned together as a group. Kids in the semi-intensive group were sent to pasture during the day and were placed in individual pens during the night. Daily nutritive requirements of all animals were determined according to daily dry matter basis as 4.3 % of live weight (NRC, 2001). Pasture quality analyses were analysed in order to determine the nutritive value of pasture during the fattening period. Botanical composition was determined according to weight by placing 1 m × 1 m cages on pasture and cutting in periods, while composition of vegetation was determined by the transect method which involves examining different 1 m × 1 cm sized lines of pasture. Thus vegetation in 100 ${\mathrm{cm}}^{2}$ for each sample was determined (Gökbulak, 2013). There was not any additional feeding for extensive group animals while the other groups were fed with lamb and calf fattening feed as concentrate feed and dry meadow grass as roughage. Feed composition given to kids during the trial is presented in Table 1.

To prevent negative effects on meat quality traits and to reduce starving stress, the last feeding was performed 12 h before slaughter. All the animals in the experimental groups were slaughtered on the same day in a private slaughterhouse close to the research farm. Following the slaughter and relevant processing on hot carcasses, the latter were placed in cold storage and chilled at +4 ${}^{\circ }C$ for 24 h. Cold carcasses were then dissected in accordance with the standard carcass jointing method developed for Mediterranean countries (Colomer-Rocher et al., 1987).

The pH was measured manually using a portable pH meter (Cyberscan PC 510 brand, pH 4.01 and 7.00 calibrated in buffer solution, USA) with a penetrating a solid type pH electrode (Sensorex, S175CD Spear Type, USA). The pH was measured immediately after carcass dressing within 45 min and after the carcasses were rested at +4 ${}^{\circ }C$ for 24 h at the same location of the longissimus dorsi (LD) muscle between the 12th and 13th thoracic vertebrae. An electrode was immersed into the muscle and kept until the values were fixed on the display screen of the pH meter, and this fixed value was read and recorded. The measurements were taken from the three different regions of the samples, and pH values were found according to the average of these values.

Meat colour ($L^{*}a^{*}b^{*}$) was evaluated 24 h post mortem on a freshly cut surface of LD muscle after 0 min and blooming in the air for 45 min using a colorimeter (Konika Minolta CR-400, Chroma Meter Reflectance) with illuminate C and 11 mm measurement diameter. The colorimeter was calibrated against a standard white plate, and three measurements were made on each sample.

The area of LD muscle cross section and a marbling score were determined at the ribbing site. Marbling is a score of intramuscular fat and evaluation of this score was made subjectively by scoring the samples according to scale prepared for marbling. To a scale from 0 to 9, no intramuscular fat was scored 0 and most intensive intramuscular fat was scored 9 (USDA, 1992; MLA, 2006).

Grau and Hamm methods were used to determine the water holding capacity (Beriain et al., 2000). The meat sample (5 g) was placed between two filter papers and then exposed to 2250 g of pressure for 5 min. Drip loss was determined according to the method of Bond and Warner (2007). The samples taken were supported by a net so that they did not come in contact with the bag, and they were kept suspended in a plastic container for 48 h at 4 ${}^{\circ }C$. After this time, the sample taken from the plastic container was weighed again after drying with drying paper. The drip loss of meat was determined as a percentage (%) by proportioning the difference between the first and the last weight measurements of the samples.

For determination of the cooking loss of meat, samples of 50 g weight were placed in vacuum bags and cooked in a hot water bath (70 ${}^{\circ }C$) for 40 min. The samples were then kept under tap water for about 30 min until they cooled to room temperature (25 ${}^{\circ }C$); then they were taken out of the bag and weighed (Mitchaothai et al., 2006). Instrumental tenderness was evaluated by a Warner–Bratzler shear force (WBSF) blade connected to Instron 3343 for texture analysis. The force applied to the meat in the Instron device was set at 50 kg and the blade speed at 200 $\mathrm{mm}\;\min^{-1}$. In texture analysis, samples used in the measurement of cooking loss was used. Three test pieces with dimensions of 1 cm × 1 cm were cut parallel to the muscle fibres from each cooked sample. The peak shear force (Shear force, $\mathrm{kgf}\;{\mathrm{cm}}^{-2}$) and force time graph obtained by measurement were recorded on the computer. Texture measurements was made at room temperature, using an aluminium P/20P probe in a texture profile analysis (TPA) device TA-XT Plus Texture Analyzer (Stable Micro Systems, Godalming, UK). Probe speed was set to 1.0 $\mathrm{mm}\;s^{-1}$, and force applying distance was set to 50 % of slice thickness. During the stiffness-of-cut test, probe speed was set to 1.0 $\mathrm{mm}\;s^{-1}$, and depth of cut was set to 75 % of slice thickness. Measurements were made as three replicates for each samples, and average values were used (Huidobro et al., 2005).

For sensory analysis, LD muscle between the 12th and 13th thoracic vertebrae was sampled. Sensory analysis was carried out in a lighted and aired room with a panelist group of 25 people. In order to reflect the possibility of sensation differences, panelists were selected between ages 25–55 with both men and women present. The panel was carried out at 16:00 local time in order to ensure panelists were neither hungry nor full, and all panelists were requested to have lunch. Panelists were told how to score and what aspects to take into consideration prior to scoring. Panelists were positioned to be uninfluential to each other, and all panelists were supplied with enough water and bread to serve as palate cleanser. All samples were presented in the same sized, shaped and coloured plates, and samples were visually identical as well. Meats were cooked with two methods (boiled and roasted). Two panel samples were prepared from one animal (boiled and roasted), and a panelist evaluated 12 samples. Panelists were requested to score a sensory assessment form including sample colour, texture, taste, smell, chewing count and general acceptance criteria (Gökalp et al., 1993; Nadarajah et al., 2005; Yaralı et al., 2013).

For approximate composition, dry matter, crude ash, intramuscular fat and protein analyses were conducted in accordance with the protocols approved by AOAC (1990). For fatty acid composition, samples taken from LD (stored at -18 ${}^{\circ }C$ for 3 months) were sent to the Food Institute of Marmara Research Center of Scientific and Technical Research Council of Turkey for relevant analyses. Analysis of fatty acids was carried out according to the method of IUPAC IID19 (1987).

Experimental data on meat quality traits and fatty acid compositions were statistically analysed (SPSS, 2005). Analyses were performed in accordance with randomized plots of 2 × 3 factorial design to determine the effects of fattening systems, breeds and interactions on investigated traits. The following model was used in analyses:

1 $$Y_{ijk}+\mu +G_{j}+{\text{BS}}_{i}+(G\times \text{BS}{)}_{ij}+\text{eijk},$$

where μ is the population mean, $G_{j}$ is the effect of the jth genotype, ${\text{BS}}_{i}$ is the effect of the ith fattening system, $(G\;\times \;\text{BS}{)}_{ij}$ is the interactive effect of the ith genotype and jth fattening system, and eijk is the random error.

**Table 2:** Meat quality traits of Hair Goat and Saanen × Hair Goat ($G_{1}$) crossbred kids.

Fattening system	Intensive fattening		Semi-intensive fattening		Extensive fattening					
Meat quality parameters	Hair Goat	Saanen × Hair Goat ($G_{1}$)	Hair Goat	Saanen × Hair Goat ($G_{1}$)	Hair Goat	Saanen × Hair Goat ($G_{1}$)	P	G	FS	G*FS
${\mathrm{pH}}_{45\;\min}$	6.40 ± 0.09	6.40 ± 0.05	6.38 ± 0.08	6.49 ± 0.03	6.36 ± 0.06	6.29 ± 0.05	–	–	–	–
${\mathrm{pH}}_{24\;h}$	5.36 ± 0.06	5.37 ± 0.02	5.44 ± 0.04	5.47 ± 0.02	5.40 ± 0.03	5.40 ± 0.04	–	–	–	–
Drip loss (%)	4.15 ± 0.25${}^{a}$	3.10 ± 0.18${}^{b}$	4.01 ± 0.44${}^{a}$	3.31 ± 0.32${}^{\text{ab}}$	3.50 ± 0.18${}^{\text{ab}}$	3.56 ± 0.29${}^{\text{ab}}$	*	*	–	*
Water holding capacity (%)	8.13 ± 0.33${}^{\text{ab}}$	8.68 ± 0.43${}^{a}$	8.06 ± 0.32${}^{\text{ab}}$	8.51 ± 0.24${}^{a}$	7.51 ± 0.24${}^{b}$	7.52 ± 0.13${}^{b}$	*	–	*	*
Cooking loss (%)	29.35 ± 1.10	29.69 ± 1.21	29.87 ± 1.00	28.58 ± 0.94	28.15 ± 0.42	28.52 ± 0.85	–	–	–	–
LD muscle area (${\mathrm{cm}}^{2}$)	13.72 ± 0.58	15.46 ± 0.40	14.16 ± 0.52	15.67 ± 0.89	13.94 ± 0.51	14.42 ± 0.48	–	–	–	–
Dry matter (%)	24.46 ± 0.49	23.15 ± 0.48	23.51 ± 0.36	23.74 ± 0.13	23.94 ± 0.25	23.92 ± 0.26	–	–	–	–
Crude ash (%)	1.09 ± 0.01${}^{\text{ab}}$	1.04 ± 0.03${}^{c}$	1.04 ± 0.01${}^{\text{bc}}$	1.10 ± 0.01${}^{a}$	1.11 ± 0.01${}^{a}$	1.12 ± 0.01${}^{a}$	**	*	–	*
Organic matter (%)	23.37 ± 0.49	22.11 ± 0.46	22.47 ± 0.36	22.64 ± 0.13	22.84 ± 0.25	22.80 ± 0.26	–	–	–	–
Crude protein (%)	25.46 ± 0.42	26.17 ± 0.49	25.38 ± 0.44	26.38 ± 0.20	25.38 ± 0.29	26.44 ± 0.41	–	–	–	–
Intramuscular fat (%)	3.01 ± 0.35${}^{a}$	2.11 ± 0.23${}^{b}$	1.82 ± 0.12${}^{\text{bc}}$	1.09 ± 0.07${}^{d}$	1.77 ± 0.11${}^{\text{bc}}$	1.32 ± 0.28${}^{\text{cd}}$	**	*	*	*
Peak shear force ($\mathrm{kgf}\;{\mathrm{cm}}^{-2}$)	5.70 ± 0.63	6.81 ± 0.78	6.60 ± 1.12	8.18 ± 1.25	7.54 ± 1.17	7.08 ± 1.11	–	–	–	–

${}^{\text{a,b,c,d}}$ The means indicated with different letters in the same row are significantly different. ${}^{*}$ P < 0.05. ${}^{**}$ P < 0.01. –: not significant. G: genotype. FS: fattening system.

## 3 Results

Meat pH has distinctive effects on colour, water holding capacity and texture, thus playing a significant role in depiction of meat quality. In the present study, meat pH values were measured at two different time periods (45 min after slaughter, ${\mathrm{pH}}_{45\;\min}$, and 24 h after slaughter, ${\mathrm{pH}}_{24\;h}$). The differences in ${\mathrm{pH}}_{45\;\min}$ and ${\mathrm{pH}}_{24\;h}$ values of the fattening systems were not found to be significant. Mean values for meat quality traits of Hair Goat and Saanen × Hair Goat ($G_{1}$) crossbred kids are provided in Table 2.

While the differences in water holding capacity and drip loss values of fattening systems were found to be significant (P < 0.05), differences in cooking loss and LD muscle area values were not found to be significant. The greatest water holding capacity was obtained from the intensive fattening system of Saanen × Hair Goat ($G_{1}$) crossbred kids, and the lowest value was obtained from the extensive fattening system of Hair Goat kids.

**Table 3:** Meat colour parameters of Hair Goat and Saanen × Hair Goat ($G_{1}$) crossbred kids.

Fattening system	Intensive fattening		Semi-intensive fattening		Extensive fattening					
Genotype	Hair Goat	Saanen × Hair Goat ($G_{1}$)	Hair Goat	Saanen × Hair Goat ($G_{1}$)	Hair Goat	Saanen × Hair Goat ($G_{1}$)	P	G	FS	G*FS
Meat colour (0th hour)										
$(L^{*}{)}^{0}$	44.23 ± 1.09${}^{\text{bc}}$	41.76 ± 1.01${}^{c}$	45.43 ± 0.86${}^{\text{ab}}$	43.51 ± 1.12${}^{\text{bc}}$	47.94 ± 1.68${}^{a}$	44.14 ± 0.98${}^{\text{bc}}$	*	–	*	*
$(a^{*}{)}^{0}$	18.62 ± 1.20	16.95 ± 0.95	18.66 ± 1.09	19.02 ± 0.79	18.93 ± 0.85	18.04 ± 1.09	–	–	–	–
$(b^{*}{)}^{0}$	9.08 ± 0.76	7.00 ± 0.69	8.36 ± 0.69	7.93 ± 0.58	9.38 ± 0.75	8.78 ± 0.84	–	–	–	–
$C^{*}$	18.86 ± 1.01	18.33 ± 0.65	20.44 ± 0.71	20.60 ± 0.69	21.12 ± 0.65	20.06 ± 0.85	–	–	–	–
$H^{*}$	25.99 ± 0.50	22.43 ± 0.57	24.13 ± 0.41	22.63 ± 0.55	26.35 ± 0.84	25.94 ± 0.75	–	–	–	–
Meat colour (45th minute)										
$(L^{*}{)}^{45}$	45.02 ± 1.25	43.21 ± 1.50	45.68 ± 0.89	44.25 ± 1.18	47.21 ± 1.78	44.93 ± 1.03	–	–	–	–
$(a^{*}{)}^{45}$	19.25 ± 1.19	18.83 ± 0.77	18.21 ± 1.15	18.58 ± 0.69	17.71 ± 0.77	18.35 ± 0.54	–	–	–	–
$(b^{*}{)}^{45}$	10.13 ± 0.85	8.10 ± 0.68	8.51 ± 0.57	8.69 ± 0.49	9.37 ± 0.79	8.75 ± 0.46	–	–	–	–
$C^{*}$	21.75 ± 1.21	19.04 ± 0.38	20.10 ± 0.35	18.81 ± 0.64	20.03 ± 0.59	20.32 ± 0.54	–	–	–	–
$H^{*}$	27.75 ± 0.65	23.29 ± 0.67	25.04 ± 0.66	25.06 ± 0.54	27.87 ± 0.88	25.49 ± 0.18	–	–	–	–
Marbling score	2.4 (1–5)${}^{a}$	2.4 (0–7)${}^{\text{ab}}$	2.1 (0–6)${}^{b}$	1.8 (0–5)${}^{c}$	2.1 (0–5)${}^{\text{ab}}$	1.5 (0–4)${}^{d}$	**	*	*	*

${}^{\text{a,b,c,d}}$ The means indicated with different letters in the same row are significantly different. ${}^{*}$ P < 0.05. ${}^{**}$ P < 0.01. –: not significant. G: genotype. FS: fattening system.

Differences in dry matter, organic matter and crude protein contents of fattening groups were not found to be significant, but the differences in intramuscular fat contents were found to be significant (P < 0.05). Intramuscular fat contents of Hair Goat kids in intensive, semi-intensive and extensive fattening systems were respectively measured as 3.01 %, 1.82 % and 1.77 %. Intramuscular fat contents of Saanen × Hair Goat ($G_{1}$) crossbred kids in intensive, semi-intensive and extensive fattening systems were respectively measured as 2.11 %, 1.09 % and 1.32 %. Meat colour parameters of lightness ($L^{*}$), redness ($a^{*}$) and yellowness ($b^{*}$) at the 0th and 45th minute are provided in Table 3. The extensive fattening group of Hair Goats had greater $L^{*}$ values.

The differences in marbling scores of fattening groups were found to be significant (P < 0.001). The intensive fattening group of Hair Goat and Saanen × Hair Goat ($G_{1}$) crossbred kids had slightly greater marbling scores than the other groups. The greatest value was obtained from the intensive fattening group of Hair Goat kids, and the lowest value was obtained from the extensive fattening group of Saanen × Hair Goat ($G_{1}$) crossbred kids.

**Table 4:** Meat fatty acid composition of Hair Goat and Saanen × Hair Goat ($G_{1}$) crossbred kids (%).

Fattening system	Intensive fattening		Semi-intensive fattening		Extensive fattening					
Fatty acids	Hair Goat	Saanen × Hair Goat ($G_{1}$)	Hair Goat	Saanen × Hair Goat ($G_{1}$)	Hair Goat	Saanen × Hair Goat ($G_{1}$)	P	G	FS	G*FS
C10:0	0.08 ± 0.006	0.09 ± 0.005	0.11 ± 0.014	0.10 ± 0.008	0.10 ± 0.010	0.10 ± 0.011	–	–	–	–
C12:0	0.05 ± 0.005	0.07 ± 0.012	0.05 ± 0.004	0.05 ± 0.004	0.06 ± 0.012	0.05 ± 0.004	–	–	–	–
C13:0	0.01 ± 0.002${}^{b}$	0,03 ± 0.009${}^{a}$	0.01 ± 0.004${}^{b}$	0.01 ± 0.000${}^{b}$	0.02 ± 0.003${}^{\text{ab}}$	0.01 ± 0.000${}^{b}$	*	*	*	*
C14:0	1.67 ± 0.12	1.91 ± 0.13	1.70 ± 0.06	1.49 ± 0.06	1.71 ± 0.15	1.68 ± 0.16	–	–	–	–
C14:1	0.08 ± 0.006	0.10 ± 0.014	0.07 ± 0.012	0.06 ± 0.011	0.08 ± 0.014	0.10 ± 0.027	–	–	–	–
C15:0	0.41 ± 0.033${}^{b}$	0.60 ± 0.056${}^{a}$	0.40 ± 0.028${}^{b}$	0.40 ± 0.052${}^{b}$	0.49 ± 0.070${}^{\text{ab}}$	0.45 ± 0.045${}^{\text{ab}}$	*	*	*	*
C16:0	20.54 ± 0.73	20.93 ± 0.40	21.31 ± 0.25	19.58 ± 0.38	20.75 ± 0.66	20.56 ± 0.82	–	–	–	–
C16:1	1.87 ± 0.06	1.99 ± 0.14	1.64 ± 0.13	1.60 ± 0.10	1.65 ± 0.06	1.70 ± 0.18	–	–	–	–
C17:0	1.65 ± 0.10	1.89 ± 0.07	1.57 ± 0.08	1.46 ± 0.20	1.61 ± 0.14	1.52 ± 0.17	–	–	–	–
C18:0	15.05 ± 1.51	16.19 ± 0.81	19.72 ± 2.33	18.14 ± 0.43	19.19 ± 1.65	18.76 ± 1.67	–	–	–	–
C18:1n9c	44.93 ± 1.72	41.01 ± 1 98	43.18 ± 2.78	43.10 ± 0.68	43.19 ± 2.03	40.63 ± 1.28	–	–	–	–
C18:2n6c	5.18 ± 1.53	3.25 ± 0.23	2.66 ± 0.28	4.15 ± 0.36	2.75 ± 0.12	5.33 ± 1.43	–	–	–	–
C18:3n3	0.11 ± 0.01${}^{b}$	0.10 ± 0.01${}^{b}$	0.25 ± 0.04${}^{a}$	0.30 ± 0.02${}^{a}$	0.24 ± 0.03${}^{a}$	0.28 ± 0.03${}^{a}$	**	–	*	*
C18:3n6	0.02 ± 0.002	0.01 ± 0.002	0.01 ± 0.000	0.01 ± 0 002	0.01 ± 0.002	0.02 ± 0.005	–	–	–	–
C20:0	0.08 ± 0.019	0.07 ± 0.010	0.07 ± 0.019	0.08 ± 0.008	0.06 ± 0.008	0.10 ± 0.020	–	–	–	–
C20:1n9c	0.09 ± 0.007	0.10 ± 0.007	0.08 ± 0.006	0.10 ± 0.007	0.08 ± 0.010	0.09 ± 0.012	–	–	–	–
C20:2	0.01 ± 0.002	0.02 ± 0.002	0.03 ± 0.018	0.03 ± 0.004	0.02 ± 0.004	0.02 ± 0.004	–	–	–	–
C20:3n6	0.01 ± 0.002${}^{b}$	0.02 ± 0.002${}^{b}$	0.01 ± 0.002${}^{b}$	0.03 ± 0.004${}^{a}$	0.02 ± 0.002${}^{b}$	0.02 ± 0.004${}^{b}$	*	*	–	*
C20:4n6	0.13 ± 0.018${}^{b}$	0.15 ± 0.025${}^{b}$	0.11 ± 0.016${}^{b}$	0.39 ± 0.092${}^{a}$	0.14 ± 0.026${}^{b}$	0.15 ± 0.043${}^{b}$	*	*	*	*
C20:5n3	1.51 ± 0.22${}^{\text{bc}}$	1.70 ± 0.02${}^{c}$	1.20 ± 0.10${}^{\text{ab}}$	1.50 ± 0.17${}^{\text{bc}}$	1.40 ± 0.03${}^{a}$	1.60 ± 0.04${}^{\text{bc}}$	*	–	*	*
C22:2	0.05 ± 0.004${}^{b}$	0.04 ± 0.007${}^{b}$	0.04 ± 0.005${}^{b}$	0.09 ± 0.018${}^{a}$	0.05 ± 0.006${}^{b}$	0.05 ± 0.010${}^{b}$	*	*	*	*
C23:0	0.01 ± 0.002	0.02 ± 0.004	0.01 ± 0.000	0.03 ± 0.010	0.01 ± 0.000	0.01 ± 0.004	–	–	–	–
CLA	0.48 ± 0.03${}^{c}$	0.52 ± 0.02${}^{c}$	0.55 ± 0.04b${}^{c}$	0.58 ± 0.05b${}^{c}$	0.65 ± 0.04${}^{\text{ab}}$	0.73 ± 0.02${}^{a}$	**	–	*	*
n-6	3.41 ± 1.53	5.33 ± 0.23	2.78 ± 0.28	4.45 ± 0.35	2.90 ± 0.10	5.50 ± 1.40	–	–	–	–
n-3	1.63 ± 0.01${}^{b}$	1.82 ± 0.01${}^{b}$	1.44 ± 0.03${}^{a}$	1.80 ± 0.02${}^{a}$	1.66 ± 0.02${}^{a}$	1.93 ± 0.03${}^{a}$	**	–	*	*
Σ SFA	19.28 ± 1.59	21.01 ± 0.76	23.75 ± 2.30	21.98 ± 0.31	23.35 ± 1.74	19.10 ± 4.04	–	–	–	–
Σ MUFA	67.30 ± 1.70	64.04 ± 1.66	66.22 ± 2.63	64.33 ± 0.54	65.72 ± 1.67	52.44 ± 10.54	–	–	–	–
Σ PUFA	5.46 ± 1.54	3.53 ± 0.26	3.06 ± 0.32	4.89 ± 0.44	3.16 ± 0.14	4.84 ± 1.53	–	–	–	–
PUFA/SFA	0.20 ± 0.03	0.16 ± 0.02	0.12 ± 0.02	0.22 ± 0.02	0.13 ± 0.02	0.25 ± 0.03	–	–	–	–
n6/n3	2.09 ± 0.55${}^{b}$	2.92 ± 0.65${}^{b}$	1.93 ± 0.45${}^{a}$	2.47 ± 0.54${}^{a}$	1.75 ± 0.14${}^{a}$	2.85 ± 0.35${}^{a}$	**	–	*	*

${}^{\text{a,b,c}}$ The means indicated with different letters in the same row are significantly different. * P < 0.05. ** P < 0.01. –: not significant. SFA: saturated fatty acids. MUFA: mono-unsaturated fatty acids. PUFA: polyunsaturated fatty acids. G: genotype. FS: fattening system.

Mean values for meat fatty acids of Hair Goat and Saanen × Hair Goat ($G_{1}$) crossbred kids in intensive, semi-intensive and extensive fattening systems are provided in Table 4. Present analyses revealed 10 saturated fatty acids (SFA), 6 monounsaturated fatty acids (MUFA) and 6 polyunsaturated fatty acids (PUFA). In all fattening groups, oleic acid was the major fatty acid in LD muscle. Also, quite high values were observed for palmitic acid and stearic acid.

The conjugated linoleic acid contents of Hair Goat kids in intensive, semi-intensive and extensive fattening systems were respectively measured as 0.48 %, 0.55 % and 0.65 %. The conjugate linoleic acid contents of Saanen × Hair Goat ($G_{1}$) crossbred kids in intensive, semi-intensive and extensive fattening systems were respectively measured as 0.52 %, 0.58 % and 0.73 %. The differences in conjugate linoleic acid contents of fattening groups were found to be significant (P < 0.001). In terms of polyunsaturated fatty acids, differences between the groups were assessed under two categories as of omega-3 and omega-6 fatty acids. Although the intensive fattening group had slightly greater omega-6 fatty acids than the other fattening groups, the differences in omega-6 fatty acids of the experimental groups were not found to be significant. The semi-intensive and extensive fattening groups had greater omega-3 fatty acids than the intensive fattening group, and the differences in omega-3 fatty acids of the experimental groups were found to be significant (P < 0.001).

**Table 5:** Sensory attributes of Hair Goat and Saanen × Hair Goat ($G_{1}$) crossbred kids.

Fattening system	Intensive fattening		Semi-intensive fattening		Extensive fattening					
Parameters	Hair Goat	Saanen × Hair Goat ($G_{1}$)	Hair Goat	Saanen × Hair Goat ($G_{1}$)	Hair Goat	Saanen × Hair Goat ($G_{1}$)	P	G	FS	G*FS
Boiled										
Colour and appearance	7.08 ± 0.22 (5–9)	6.88 ± 0.30 (4–9)	7.12 ± 0.19 (6–9)	6.96 ± 0.22 (5–9)	7.16 ± 0.24 (4–9)	7.16 ± 0.21 (5–9)	–	–	–	–
Smell density	7.20 ± 0.19 (5–9)	7.12 ± 0.25 (4–9)	7.12 ± 0.19 (5–9)	7.28 ± 0.19 (5–9)	7.00 ± 0.18 (6–9)	7.20 ± 0.23 (5–9)	–	–	–	–
Taste/flavour	7.04 ± 0.24 (4–9)	7.32 ± 0.28 (4–9)	7.12 ± 0.20 (4–9)	6.92 ± 0.22 (4–9)	7.36 ± 0.22 (4–9)	6.84 ± 0.26 (3–9)	–	–	–	–
Texture	7.08 ± 0.25 (3–9)	7.24 ± 0.27 (5–9)	7.28 ± 0.20 (6–9)	7.04 ± 0.22 (5–9)	7.36 ± 0.22 (5–9)	7.12 ± 0.19 (6–9)	–	–	–	–
General taste	7.10 ± 0.19	7.14 ± 0.24	7.16 ± 0.15	7.05 ± 0.18	7.22 ± 0.19	7.08 ± 0.18	–	–	–	–
Roasted										
Colour and appearance	6.84 ± 0.26 (4–9)	6.88 ± 0.26 (4–9)	6.92 ± 0.21 (5–9)	7.12 ± 0.19 (5–9)	7.32 ± 0.17 (5–9)	6.96 ± 0.23 (5–9)	–	–	–	–
Smell density	7.00 ± 0.24 (4–9)	7.24 ± 0.24 (4–9)	7.12 ± 0.25 (5–9)	7.40 ± 0.17 (6–9)	7.28 ± 0.17 (6–9)	7.20 ± 0.21 (4–9)	–	–	–	–
Taste/flavour	6.84 ± 0.27 (4–9)	7.16 ± 0.24 (4–9)	7.28 ± 0.22 (5–9)	7.04 ± 0.25 (2–8)	7.24 ± 0.23 (3–9)	6.96 ± 0.20 (4–8)	–	–	–	–
Texture	6.76 ± 0.28 (4–9)	6.88 ± 0.23 (5–9)	7.60 ± 0.21 (6–9)	7.12 ± 0.21 (4–9)	7.56 ± 0.16 (6–9)	7.08 ± 0.15 (6–9)	–	–	–	–
General taste	6.86 ± 0.24	7.04 ± 0.22	7.23 ± 0.18	7.17 ± 0.15	7.35 ± 0.14	7.05 ± 0.15	–	–	–	–

Mean values for meat sensory attributes assigned by taste panel are provided in Table 5. Evaluations were made of the differences in sensory attributes based on animal breeds and cooking method. Panelists assigned similar scores for colour/appearance, smell density, taste/flavour, and texture of boiled and roasted meat of Hair Goat and Saanen × Hair Goat ($G_{1}$) crossbred kids fattened in intensive, semi-intensive and extensive fattening systems.

**Table 6:** Texture profile analysis of Hair Goat and Saanen × Hair Goat ($G_{1}$) crossbred kids.

Fattening system	Intensive fattening		Semi-intensive fattening		Extensive fattening					
Meat quality parameters	Hair Goat	Saanen × Hair Goat ($G_{1}$)	Hair Goat	Saanen × Hair Goat ($G_{1}$)	Hair Goat	Saanen × Hair Goat ($G_{1}$)	P	G	FS	G*FS
Hardness ($N\;{\mathrm{cm}}^{-2}$)	60.21 ± 8.20${}^{\text{cd}}$	44.97 ± 4.30${}^{d}$	88.98 ± 11.44${}^{b}$	70.67 ± 9.94${}^{\text{bcd}}$	75.06 ± 7.48${}^{\text{bc}}$	94.48 ± 10.34${}^{a}$	**	**	–	–
Adhesiveness ($N\;s^{-1}$)	-0.06±0.02	-0.04±0.01	-0.03±0.01	-0.06±0.01	-0.09±0.03	-0.04±0.02	–	–	–	–
Springiness (cm)	0.80 ± 0.01${}^{\text{ab}}$	0.74 ± 0.01${}^{c}$	0.86 ± 0.03${}^{a}$	0.77 ± 0.02${}^{\text{bc}}$	0.78 ± 0.03${}^{\text{bc}}$	0.80 ± 0.01${}^{\text{ab}}$	*	*	–	–
Cohesiveness (ratio)	0.72 ± 0.01${}^{a}$	0.63 ± 0.02${}^{b}$	0.72 ± 0.01${}^{a}$	0.69 ± 0.01${}^{a}$	0.68 ± 0.01${}^{a}$	0.70 ± 0.01${}^{a}$	**	–	*	*
Gumminess ($N\;{\mathrm{cm}}^{-2}$)	42.90 ± 5.57${}^{\text{cd}}$	28.36 ± 2.83${}^{d}$	63.63 ± 8.03${}^{\text{ab}}$	48.76 ± 6.82${}^{\text{bc}}$	51.13 ± 6.00${}^{\text{bc}}$	80.4 ± 7.54${}^{a}$	**	*	–	*
Chewiness ($N\;{\mathrm{cm}}^{-1}$)	34.33 ± 4.36${}^{\text{cd}}$	21.10 ± 2.29${}^{d}$	54.86 ± 7.42${}^{\text{ab}}$	37.73 ± 5.91${}^{c}$	40.35 ± 4.83${}^{\text{bc}}$	64.61 ± 6.34${}^{a}$	**	**	–	*
Resilience	0.36 ± 0.01${}^{a}$	0.28 ± 0.01${}^{c}$	0.35 ± 0.01${}^{\text{ab}}$	0.33 ± 0.01${}^{b}$	0.33 ± 0.01${}^{b}$	0.35 ± 0.01${}^{\text{ab}}$	**	*	–	–

${}^{\text{a,b,c,d}}$ The means indicated with different letters in the same row are significantly different. ${}^{*}$ P < 0.05. ${}^{**}$ P < 0.01. –: not significant. G: genotype. FS: fattening system.

Texture profile analysis of meat samples was conducted and results are provided in Table 6. The differences in hardness, adhesiveness, springiness, cohesiveness, gumminess, chewiness and resilience values of the experimental groups were found to be significant. Meat hardness values varied between 94.48–44.97 $N\;{\mathrm{cm}}^{-2}$; gumminess values varied between 80.4–28.36 $N\;{\mathrm{cm}}^{-2}$, chewiness values varied between 64.61–21.10 $N\;{\mathrm{cm}}^{-1}$ and the greatest values were obtained from extensive fattening group of Saanen × Hair Goat ($G_{1}$) crossbred kids. Meat springiness values varied between 0.86–0.74, cohesiveness values varied between 0.72–0.63 and resilience values varied between 0.36–0.28.

## 4 Discussion

Recent research has mostly focused on improvement of meat yield and quality. Consumers largely prefer visually appealing meat served on market shelves. Meat quality is largely influenced by several factors including meat fat and fat colour, intermuscular fat ratio, marbling degree, texture, water holding capacity, sensory attributes, food security, animal welfare, taste, and growing and fattening conditions (Thu, 2006; Warner et al., 2010).

Following the slaughter, increasing acidity, decreasing pH and final pH values play a great role in technological processes to be applied to preserve the qualitative and microbiological characteristics of fresh meat. The pH is among the most significant parameters used to assess postmortem changes encountered in carcasses. Muscle pH of livestock varies between 7.0–7.3. Following the slaughter, pH values decrease slightly below 7.0 within 40 min and vary between 5.36–5.47 within 24 h, and no further decrease is seen in meat pH values (Simek et al., 2003; Sen et al., 2004; Sanudo et al., 2007; Madruga et al., 2008; Yagoubi et al., 2018).

Water holding capacity is another factor designating meat quality. Water holding capacity is defined as water-bonding capability of muscle or muscle products under various conditions. This attribute influences both qualitative (vitamin, mineral, salt holding) and quantitative (water holding) traits of fresh meat and meat products (Abdullah and Musallam, 2007; Sanudo et al., 2007). Some studies indicate that breed and slaughter weight have an effect on water holding capacity, while some studies report that it is not. In addition, it is stated that animals fed with high protein rations have high water holding capacity, and meats with high water holding capacity will be more juicy and delicious. In the study, the differences in water holding capacity and drip loss values of fattening groups were found to be significant (P < 0.05); the greatest water holding capacity was obtained from the intensive fattening group of Saanen × Hair Goat ($G_{1}$) crossbred kids, and the lowest value was obtained from the extensive fattening group of Hair Goat kids. Although significant effects of fattening systems on water holding capacity were reported in some studies, there are some other studies reporting lower water holding capacity for animals fattened in pasture (Diaz et al., 2002; Santos-Silva et al., 2002).

LD muscle area is an important indicator of carcass development. The greatest LD muscle area was obtained from the intensive fattening group of Saanen × Hair Goat ($G_{1}$) crossbred kids. Since this group had the greatest final live weight at the end of fattening period, it was usual to have the greatest LD muscle area from this group.

Tissue myoglobin concentration designates the meat colour, and such a value may vary with the species, breed, gender, age, type of muscle and physical activity (Young and West, 2001). Today, the primary target of the meat industry is to produce meat with optimum colour parameters (Mancini and Hunt, 2005). Colour is the most significant sensory attribute influencing acceptability of meat and meat products. Consumers generally relate colour with freshness, taste, crispness, reliability, storage duration and nutritional attributes of meat and thus have made colour as an important purchase criterion. Present values measured at the 0th and 45th minute revealed that the extensive fattening group of Hair Goat kids had greater lightness ($L^{*}$) values, and the intensive fattening group of Hair Goat kids had greater redness ($a^{*}$) values. It was indicated in previous studies that increasing pH values darkened meat colour, and decreasing pH values increased the lightness of meat (Young and West, 2001; Dhanda et al., 2003; Lawrie and Ledward, 2006; Sanudo et al., 2007). Present findings on meat colour parameters comply with these literature findings.

Marbling is defined as fat intrusion into muscle fibres and formation of a mosaic-like structure. Present findings revealed that the intensive fattening group of Hair Goat and Saanen × Hair Goat ($G_{1}$) crossbred kids had greater marbling scores than the other groups. The greatest marbling value was obtained from the intensive fattening group of Hair Goat kids and the lowest value from the extensive fattening group of Saanen × Hair Goat ($G_{1}$) crossbred kids. It was seen that intermuscular fat deposition was better in the intensive fattening group and Hair Goat kids.

Fatty acids influence various meat quality traits including hardness, firmness, aroma and shelf life. Fatty acid composition and different melting points of fatty acids directly influence meat hardness and firmness. Volatility of fatty acids and cooking-induced oxidation products influence meat aroma (Yaralı et al., 2007). In the present study, 10 SFA, 6 MUFA and 6 PUFA were identified in meat samples. Oleic acid was the major fatty acid in LD tissue of all groups. High quantities of palmitic acid and stearic acid were also encountered in meat samples. Previous studies also reported that oleic acid was the major fatty acid in LD tissue fatty acid composition of different goat breeds and it was followed by palmitic and stearic acid (Santos et al., 2007; Peña et al., 2009; Karaca, 2010).

Studies show that the amount of conjugated linoleic acid in meat increases linearly with the increase in the ratio of green feed in the ration and in the ratio of roughage. The differences in conjugated linoleic acid content between the groups were found to be significant in the study (P < 0.001). The extensive fattening group of Hair Goat and Saanen × Hari goat ($G_{1}$) crossbred kids had greater conjugate linoleic acid contents than the other groups. In both genotypes, conjugate linoleic acid contents increased from intensive fattening toward extensive fattening. Previous studies also reported significant effects of feeding on meat conjugate linoleic acid contents (Santos-Silva et al., 2002; Piasentier, 2003; Nuernberg et al., 2008; Yaralı, 2010).

The differences in total SFA, total MUFA and total PUFA of Hair Goat and Saanen × Hair Goat ($G_{1}$) crossbred kids fattened in intensive, semi-intensive and extensive fattening systems were not found to be significant. However, some researchers reported significant effects of kid genotype on SFA contents (Sikora and Borys, 2006; Horcada et al., 2012). However, similar to present findings, some others reported insignificant effects of genotypes on SFA contents of goat meat (Stankov et al., 2002; Ekiz et al., 2014). Complying with the present findings, previous researchers also reported insignificant effects of genotype on total MUFA contents (Brzostowski et al., 2008; Peña et al., 2009). Present total PUFA contents comply with the literature findings (Karaca, 2010; Ekiz et al., 2014).

Polyunsaturated fatty acids were assessed over omega-3 and omega-6 fatty acids. Omega-3 fatty acids increased in all genotypes from intensive fattening toward to extensive fattening. Such a case indicated that omega-3 fatty acids increased with increasing animal benefit from pasture. Considering the genotypes, it was observed that Saanen × Hair Goat ($G_{1}$) crossbred kids had greater omega-6 contents in all fattening systems. Parallel to present findings, it was reported in previous studies that omega-6 fatty acids did not change with the fattening systems (Valesco et al., 2000; Nuernberg et al., 2008). It was observed that the omega-3 values were different between the fattening groups, and such values were found to be significant (P < 0.05). Present findings on omega-3 fatty acids (C18:3, C20:5 and C22:6) complied with the findings of previous studies conducted with goat kids (Rhee et al., 2000; Lee et al., 2008).

Sensory tests can be conducted on meat and meat products at any time from the slaughter until the consumption. Biochemical reactions encountered during the meat ageing may influence meat quality. These reactions generally influence meat colour, hardness and juiciness (Nute, 2002; Liu et al., 2003). According to scores assigned by taste panel, differences in sensory attributes of Hair Goat and Saanen × Hair Goat ($G_{1}$) crossbred kids were not found to be significant. Present findings partially comply with the results of a previous study conducted with Hair Goats fattened in different systems (Karaca, 2010). However, several studies reported significant differences in sensory attributes of goat meat based on breeds and fattening systems (Yılmaz et al., 2009; Ekiz et al., 2010; Yalçıntan, 2011).

Texture covers hardness, firmness, cohesiveness, crispness and dispersibility of a foodstuff (Szczesniak, 2002). There are two common methods used to measure texture of foodstuffs: the Warner–Bratzler method and texture profile analysis (TPA) (Culloli, 1995). In texture profile analysis, various values are recorded while a physical force was exerted on the product. With these analyses, hardness, chewiness and springiness attributes are tested (Ruiz de Huidobro et al., 2001; Huidobro et al., 2005). Considering the peak shear force of the meat samples, it was observed that the semi-intensive group of Saanen × Hair Goat ($G_{1}$) crossbred kids had greater values than the other groups. In previous studies, the differences in peak shear force of experimental groups fed with mixed rations and fed in pasture were not found to be significant, but pasture-fed lambs had harder meats (Santos-Silva et al., 2002; Carrasco et al., 2009).

Textural attributes are related to fat, salt and pH values, and these parameters play a great role in consumer preferences (Gökalp et al., 2010). Fats have significant functions in identification of appearance (colour, lightness), texture (viscosity, elasticity and hardness), taste and mouthfeel (melting, slickness) (Crehan et al., 2000; Garcia et al., 2002; Sampaio et al., 2004). Present findings on textural attributes revealed that hardness, gumminess and chewability values increased with decreasing fat quantities of the samples. Hardness, chewability and elasticity are important parameters used in the assessment of meat texture (Ekiz et al., 2010). Present hardness values varied between 94.48–44.97 $N\;{\mathrm{cm}}^{-2}$ with the greatest value from the intensive fattening group of Saanen × Hair Goat ($G_{1}$) crossbred kids and the lowest value from the intensive fattening group of Hair Goat kids. It was also observed that the intensive fattening group of Saanen × Hair Goat ($G_{1}$) crossbred kids with a low marbling score had the greatest gumminess (80.4 N) and chewability (64.61 N) values.

Sheep and goat breeding in our country is an indispensable sector which has important roles in nutrition, providing employment and development of provinces. Although current sheep and goat breeding is being practised by traditional methods in suburban areas with native breeds which have low productivity, efforts are being made to solve these issues with genetical improvement studies, new nutrition strategies and improving agricultural systems. There are a limited number of fattening studies with kids in our country, and this is dependent on market demands. In recent years there is increasing concern about fats and fatty acids in meat and meat products. Production systems are very important for both product yield and quality and production economy. Nutrition systems are the most studied area for fatty acid composition. In this study on the effects of different nutrition methods on meat quality and fatty acids composition, it was determined that water holding capacity of meat was higher in the intensive group, while meat colour was brighter in the Hair Goat group, and there was no differences in nutrient contents except for fatty acids between groups. When the study is evaluated as a whole, it can be said that the semi-intensive fattening method is more suitable for both genotypes. The conjugated linoleic acid ratio and omega-3 fatty acids were higher for the extensive group compared to the intensive group. More studies in this area are needed to evaluate these data better. It could be possible to increase our comprehension of the productivity of the Hair Goat breed and its crossbreed genotypes with more studies on different productive systems.

## Data Availability

Data are available from the corresponding author upon request.
